# A transformed global enterprise for an HIV vaccine

**DOI:** 10.1002/jia2.25822

**Published:** 2021-11-21

**Authors:** Roger Tatoud, William Snow, José Esparza

**Affiliations:** ^1^ International AIDS Society Geneva Switzerland; ^2^ Forestville California USA; ^3^ University of Maryland Baltimore Maryland USA

**Keywords:** biomedical research, COVID‐19, HIV, public–private sector partnerships, vaccine

The COVID‐19 pandemic has shown that the global scientific community, public and private funders, as well as governments, can come together to develop safe and effective vaccines in record time. Success was in large part due to the focus on harnessing existing and new science to develop a vaccine urgently needed for global public health use. That sense of urgency, as well as proof‐of‐concept that a vaccine was possible, drove the rapid discovery of COVID‐19 vaccines [1, 2].

Scientific advances in one field are often nurtured by previous research in other fields and progress in one area can reinvigorate research and development (R&D) in another. In many ways, the COVID‐19 vaccine success builds on 40 years of HIV vaccine R&D, including basic science, vaccine platforms, clinical experience and networks, and close interactions between scientists and affected communities. HIV vaccine scientists quickly pivoted, or expanded their work, to COVID‐19 vaccine R&D [3]. Beyond science, funding matched the task. Pull mechanisms, which foster demand for an undeveloped product, complemented push mechanisms, which lower the cost of R&D to fund targeted development projects and transnational partnerships. Unlike COVID‐19, where science was not the main challenge, HIV vaccine development remains at a discovery stage, and the creation of a new system of incentives to mirror what is unfolding in the COVID‐19 response [4] could enhance the global research effort.

In this viewpoint, we revisit the early days of the Global HIV Vaccine Enterprise (the Enterprise) and explore how the COVID‐19 vaccine development effort could inform a future transformation of an organization that has supported the vaccine R&D field since 2005 (Table 1), first as an alliance of stakeholders, and now as a convener hosted at the IAS – the International AIDS Society.

**Table 1:** Global HIV Vaccine Enterprise achievements and key clinical efficacy trials

Year	Achievements and key clinical efficacy trials
2003	Global HIV Vaccine Enterprise concept first published in Science Ref. [5]. AIDSVAX® VAX003 and VAX004 clinical trials show no efficacy for the prevention of acquisition or for modification of HIV infection.
2004	The G8 at the Sea Island summit endorses and establishes the Global HIV Vaccine Enterprise. Interim secretariat of the Enterprise established at the Bill & Melinda Gates Foundation.
2005	The Global HIV/AIDS Vaccine Enterprise: scientific strategic plan published in PLoS Medicine Ref. [6]. First stakeholders meeting held at the Wellcome Trust in London, UK, followed by a Funder's forum. Establishment by the NIH of the Center for HIV/AIDS Vaccine Immunology (CHAVI) first implementation projects under the Global HIV Vaccine Enterprise.
	Establishment by the Bill and Melinda Gates Foundation of the Collaboration for AIDS Vaccine Discovery (CAVD), second implementation projects under the Global HIV Vaccine Enterprise.
2007	WHO‐UNAIDS, AVAC and the Enterprise form a coordinating group and communication subgroup.
2008	First executive director appointed, and secretariat established in New York, USA. Enterprise launched the “New minds new ideas” to attract the next generation of investigators and technologies to HIV vaccine research (AIDS 2008 Conference in Mexico City, Mexico).
2009	Enterprise Science Committee established to discuss the state of the field. The AIDS Vaccine for Asia Network (AVAN) created following a consultation with WHO‐UNAIDS in Beijing.
2010	Publication of the 2010 scientific strategic plan of the Global HIV Vaccine Enterprise in Nature Medicine Ref. [7]. Enterprise, WHO, UNAIDS and the US–MHRP convene a meeting to develop recommendations on the future of RV144.
2011	Enterprise board identifies convening, funding and collaboration as top priorities, re‐iterating the concept of preparing comprehensive scientific strategic plans.
2012	Enterprise secretariat takes up residence at IAVI headquarter in New York under a new director. Enterprise launches the Timely Topics to discuss cross cutting themes proposed by investigators.
2013	13th and last AIDS Vaccine Conference, Barcelona, Spain – October 7–10, marking the transition to the biannual “HIV Research for Prevention” (R4P) Conference, the only global scientific conference focused exclusively on the challenging and fast‐growing field of HIV prevention research. Launch of the Bench to Clinic webtool. HVTN 505 DNA plus protein efficacy trial stopped due to lack of efficacy.
2014	First Research for Prevention Conference led by Enterprise held in Cape Town, South Africa.
2015	Enterprise holds a Product Development Bootcamp after creating a guidance document on Target Product Profile for a preventive HIV Vaccine. European AIDS Vaccine Initiative (EAVI2020) and the European HIV Vaccine Alliance (EHVA) are funded by the European Commission to foster a multidisciplinary approach to HIV vaccine development.
2016	HVTN 702 (Uhambo) launched to test whether a new version of the RV144 HIV vaccine candidate safely prevents HIV infection among adults in South Africa.
2017	Launched of the HVTN 705 (Imbokodo) trial, a Phase 2b proof‐of‐concept study in HIV‐negative women in Africa evaluating the safety and efficacy of an experimental regimen based on a “mosaic” vaccine designed to induce immune responses against a wide variety of global HIV strains.
2018	Enterprise transfers to the International AIDS Society (IAS). Global HIV/AIDS Vaccine Enterprise Strategic Plan 2018–2023 presented at the Research for Prevention Conference, Madrid, Spain. Launch of PrEPVacc, an African‐led, European‐supported HIV prevention study running in eastern and southern Africa from 2018 to 2023. For the first time, a study will evaluate an experimental HIV vaccine and pre‐exposure prophylaxis (PrEP) at the same time.
2019	Launched of the HVTN 706 (MOSAICO), a trial using products similar to HVTN 705 recruiting HIV‐negative men and transgender people who have sex with men in the Americas and Europe. Enterprise at the IAS convenes its first stakeholder meeting at the 10th AIDS Conference in Mexico City, Mexico, followed by a convening of EU stakeholders in Ghent, Belgium, an opportunity to engage as a region on critical issues, including political and research commitments in Europe.
2020	HVTN 702 discontinued for futility. The SARS‐CoV2 pandemic lead to global lockdowns. Enterprise holds online events updating stakeholders on HIV vaccine R&D, preparing the field for result of ongoing efficacy trials, supporting new research into design for efficacy trials in a fast‐changing prevention landscape and advocating for sustained investment in vaccine R&D. On 11 December 2020, the U.S. Food and Drug Administration issued the first emergency use authorization (EUA) for a vaccine for the prevention of coronavirus disease 2019.
2021	HVTN 705 (Imbokodo) discontinued for lack of efficacy.

Abbreviations: AVAC, AIDS Vaccine Advocacy Coalition; NIH, National Institutes of Health; UNAIDS, The Joint United Nations Programme on HIV/AIDS; R&D, reseach and development; US‐MHRP, U.S. Military HIV Research Program; WHO, World Health Organization.

At the turn of the 21st century, after 20 years of mostly investigator‐driven HIV vaccine research and realizing the complexity of the challenges confronting the scientific community, it became clear that a more coordinated and collaborative effort was needed to accelerate HIV vaccine development [5]. Consequently, the Global HIV Vaccine Enterprise was established in 2005 [6]. Its initial impulse was to identify gaps in research or areas in need of better collaboration, from basic immunology to conduct of clinical trials. The expectation was that a scientific plan developed collaboratively would serve as a roadmap for the scientific community and funders [7]. Much new science was produced under the plan [8], but it was not always enough to convince scientists, donors and industry that we knew enough to proceed energetically with proof‐of‐concept clinical trials of a variety of candidate vaccines. Largely, it was felt that the best use of existing funding was still to solve basic scientific questions, rather than empirically conduct trials.

In 2018, the Enterprise evolved into a convening enabler with a strategy to maintain momentum for scientific collaboration while orienting the vaccine community around emerging priorities in late‐stage product development [9]. Many scientific and operational challenges that hindered the development of a vaccine in 2005 remain unsolved today [10]. As we emerge from the COVID‐19 pandemic, and with an HIV prevention landscape evolving quickly, the times are propitious to renew the mission of the Enterprise.

A rejuvenated Enterprise can provide the impetus for making a case for more empirical research, be a place to identify and support exploration of out‐of‐the‐paradigm approaches and a forum to connect innovators, researchers, the pharmaceutical industry, regulators, funders and communities to explore new ways of working, stimulate coordinated long‐term innovative funding strategies and foster greater dialogue between funders and researchers [11].

An HIV vaccine is still needed – and still achievable [12]. New exciting HIV vaccine science continue to be produced, strengthening our conviction that we will be able to create a vaccine. But exploratory science alone will not be sufficient to develop a vaccine, and a long‐term, end‐to‐end strategy of product development which includes a systematic and coordinated pipeline of vaccine constructs that can be selected, tested, evaluated, refined – and evaluated in parallel rather than sequentially could accelerate the development of a vaccine.

Vaccine development challenges addressed collectively can lead to solutions applied collectively. Meaningful collaborations that have delivered on challenges, such as development of broadly neutralizing antibodies, must be extended to incorporate product development and evaluation. Renewed and expanded collaborations that would emulate what COVAX, the vaccines pillar of the Access to COVID‐19 Tools (ACT) Accelerator and the US Operation Warp Speed have done fast and well will benefit from the Enterprise as a convener.

Looking forward, *sustainable strategic networks* should include industry, funders and, importantly work towards effective coordination among stakeholders to promote long‐term strategic cooperation [13]. Such networks must include researchers worldwide as the balance of expertise and funding still leans heavily towards the global North, a dominance that was recognized as early as 2004 when the G8 provided support for a global vaccine enterprise [14]. It is vital to ensure that most‐affected communities are included in the research, which would improve demand for and acceptance of vaccines. The Enterprise can also support the role of national, regional and global organizations (including WHO and UNAIDS) that have associated aims.

Finally, in addition to new and diversified investment, the Enterprise could be an attractive go‐between to bring back more industries to the table. New incentives could de‐risk, scientifically and financially, involvement of the private sector, which has patiently waited for the scientific community for reassurance that basic scientific problems in development of an HIV vaccine have been solved. This is probably one of the major lessons from the COVID‐19 vaccine experience; public–private partnerships can be very powerful [15].

As envisaged in 2005, the Enterprise should remain a forum where partners can share their vision, build and plan collaborative efforts. To succeed, the Enterprise needs to be given the mandate and means to engage with stakeholders to deliver on a challenge with greater complexity than COVID‐19 vaccine development. Building on a leadership that has been at the centre of the COVID‐19 vaccine success, it is realistic that the Enterprise could emulate the COVID‐19 success. As originally envisioned: “The Enterprise would be a steering, coordinating and monitoring organization with an emphais on openness, collegiality, data sharing and commitment”. The early discontinuation of the HVTN 702 study (which followed in the steps of the RV144, the only vaccine efficacy study that ever showed a positive, although modest protection), a pandemic of worldwide consequences and progress in HIV prevention strengthen the case for a new beginning for the Global Vaccine Enterprise.

The challenge will be to build consensus for change in the HIV vaccine R&D field, which many have started to acknowledge is crucial, as they have learned from COVID‐19 [16]. We need to make HIV vaccine research a public health priority, as was done for COVID‐19. In this changing global health landscape, the COVID‐19 pandemic may be the *black swan* [17] that will give birth to a transformed global enterprise for an HIV vaccine. Our collective challenge and responsibility are to find the way to realistically implement a shared vision of a world where the HIV epidemic is finally fully controlled.

## COMPETING INTERESTS

None to declare.

## AUTHORS’ CONTRIBUTIONS

RT, WS and JE contributed equally to the production of this manuscript.

## FUNDING

None.

## DISCLAIMER

The views expressed in this document are those of the authors alone and may not in any circumstances be regarded as stating an official position.
